# Synergistic induction of PGE2 by oral pathogens and TNF promotes gingival fibroblast-driven stromal-immune cross-talk in periodontitis

**DOI:** 10.1128/mbio.00046-25

**Published:** 2025-04-03

**Authors:** Elwira Nieboga, Aureliusz Schuster, Dominika M. Drapala, Mariia Melnykova, Aleksander Gut, Weronika Lipska, Mateusz Kwitniewski, Marcin Migaczewski, Marta Czesnikiewicz-Guzik, Tomasz Kaczmarzyk, Jan Potempa, Aleksander M. Grabiec

**Affiliations:** 1 Department of Microbiology, Faculty of Biochemistry, Biophysics and Biotechnology, Jagiellonian University, Kraków, Poland; 2 Doctoral School of Exact and Natural Sciences, Jagiellonian University, Kraków, Poland; 3 Chair of Oral Surgery, Faculty of Medicine, Jagiellonian University Medical College, Kraków, Poland; 4 Department of Periodontology, Preventive Dentistry and Oral Medicine, Faculty of Medicine, Jagiellonian University Medical College, Kraków, Poland; 5 Department of Immunology, Faculty of Biochemistry, Biophysics and Biotechnology, Jagiellonian University, Kraków, Poland; 6 2nd Department of General Surgery, Jagiellonian University Medical College, Kraków, Poland; 7 Oral Sciences, University of Glasgow Dental School, School of Medicine, Dentistry & Nursing, College of Medical, Veterinary and Life Sciences, University of Glasgow 12194 , Glasgow, United Kingdom; 8 Department of Oral Immunology and Infectious Diseases, University of Louisville School of Dentistry 162193, Louisville, Kentucky, USA; Georgia Institute of Technology, Atlanta, Georgia, USA

**Keywords:** periodontitis, host-pathogen interactions, *Porphyromonas gingivalis*, prostaglandin, inflammation

## Abstract

The interaction between pathogenic microorganisms and stromal cells, in particular fibroblasts, significantly contributes to the pathogenesis of many bacterially driven diseases. In periodontitis, oral pathogens penetrate the epithelial barrier and aggravate ongoing gingival inflammation by promoting the production of inflammatory mediators, such as prostaglandin E2 (PGE2). This study aimed to investigate the functional consequences of the interplay between oral pathogens and a pro-inflammatory environment in the activation of the PGE2 pathway in primary human gingival fibroblasts (GFs). GF infection with *Fusobacterium nucleatum*, *Porphyromonas gingivalis,* or *Filifactor alocis* in the presence of tumor necrosis factor (TNF) led to synergistic induction of cyclooxygenase-2 (COX-2), a key enzyme in the PGE2 synthesis pathway, as well as secretion of PGE2. A similar synergy in COX-2 upregulation was observed upon GF infection with oral pathogens in the presence of IL-1α, IL-1β, and interferon-α (IFN-α). This effect required toll-like receptor-2 (TLR2) and the p38 MAP kinase activation and was specific for fibroblasts as infection of macrophages or keratinocytes with oral pathogens in the proinflammatory environment did not cause synergistic COX-2 induction. Finally, we demonstrated that conditioned media from GFs infected with *F. nucleatum* under inflammatory conditions amplified the expression of the neutrophil chemokine *IL8* in macrophages and confirmed that this effect was mediated by synergistic induction of PGE2 in GFs. Collectively, we identify a new mechanism of stromal-immune cross-talk that is driven by synergistic PGE2 induction by oral pathogens and inflammatory cytokines in GFs and may contribute to excessive macrophage activation and neutrophil infiltration in periodontitis.

**IMPORTANCE:**

Periodontitis is a highly prevalent, dysbiosis-driven chronic inflammatory disease that not only leads to tooth loss but also is associated with severe systemic diseases. In this work, we describe a novel mechanism responsible for excessive production of PGE2, which is a potent inflammatory mediator that significantly contributes to the pathogenesis of periodontitis. We found that infection of GFs with many species of oral pathogens in the presence of inflammatory cytokines produced by the host leads to synergistic induction of COX-2 expression and PGE2 production. We found that this fibroblast-specific amplification of the COX-2-PGE2 axis by oral pathogens and cytokines is driven by the p38 MAP kinase and promotes enhanced expression of a key neutrophil chemokine by macrophages. These studies have thus enabled the identification of a new mechanism of host-pathogen interactions in periodontitis, improving our understanding of the roles of GFs and their cross-talk with immune cells in disease pathogenesis.

## INTRODUCTION

Within the oral mucosa, fibroblasts are one of the most abundant cell populations that not only maintain tissue structure and integrity but also act as sentinel cells displaying high-level inflammatory responsiveness (1). The immune functionality of gingival fibroblasts (GFs) is evident in periodontitis, a chronic inflammatory disease of the periodontium which, in its severe form, affects more than 10% of the adult population. Periodontitis not only leads to tooth loss but also promotes the development of many systemic diseases, including rheumatoid arthritis, Alzheimer’s disease, cardiovascular disease, diabetes, and cancer (2). It is now accepted that the disease is initiated by microbial imbalance (dysbiosis) that drives the non-resolving activation of the immune system converging on the recruitment of excessive numbers of activated neutrophils that are responsible for a significant amount of periodontal tissue damage (2). While many immune and non-immune cell types release neutrophil-attracting mediators, recent insights from single-cell transcriptomic studies identified the specific GF hyperresponsiveness toward recruitment of neutrophils (3), confirming the key role of the fibroblast-neutrophil cross-talk in the immunopathology of periodontitis.

Prostaglandin E2 (PGE2) is a lipid mediator released by many cell types, including GFs, that significantly contributes to the pathobiology of periodontitis (4). In the process of PGE2 biosynthesis, arachidonic acid is initially converted to prostaglandin H2 (PGH2) by cyclooxygenases (the constitutively expressed COX-1 and the inducible COX-2) which is followed by PGH2 conversion to PGE2 by PGE synthases, in particular microsomal prostaglandin E synthase-1 (mPGES-1). Although PGE2 is commonly considered an inflammatory mediator associated with acute inflammation, accumulating evidence suggests its prominent role in chronic inflammatory diseases (5). Indeed, COX-2 expression is not restricted to sites of acute inflammation—it is readily detected in tissues affected by chronic inflammation such as synovial tissue in rheumatoid arthritis, the colon in inflammatory bowel disease, and the spinal cord in multiple sclerosis (5). COX-2 levels are also elevated in gingival tissue from periodontitis patients compared to controls (6, 7) and, consistently, increased PGE2 concentrations are observed in gingival crevicular fluid (GCF) from patients with periodontitis, correlating with gingival inflammation and clinical parameters (8–10). Of note, GCF PGE2 levels are significantly reduced after periodontal therapy (9, 10). In addition, polymorphisms in the *PTGS2* gene encoding COX-2 and alterations in the methylation levels in the *PTGS2* promoter have been linked to periodontitis (11, 12). In animal models, the application of a specific antagonist of the PGE2 receptor EP4, COX-2 inhibitors with distinct selectivity profiles, or an mPGES-1 inhibitor uniformly protects against alveolar bone loss, possibly through inhibition of osteoclastogenesis (13–15).

In GFs, COX-2 expression and PGE2 production are upregulated by multiple factors associated with periodontitis pathogenesis, including inflammatory cytokines (16), oral pathogens, such as *Porphyromonas gingivalis* and *Filifactor alocis* (17, 18), or bacterial virulence factors, in particular, lipopolysaccharides (LPS) and *P. gingivalis*-derived fimbriae (19, 20). However, studies of GF responses that are restricted to exposure to a single factor might not reflect the complexity of cell responses to the combination of multiple host- and bacteria-derived inflammatory stimuli present in the inflamed gingival tissue and could underestimate the true scope of COX-2 and PGE2 regulation. Consistent with this notion, it has been demonstrated that LPS from *P. gingivalis*, which alone is a weak activator of GFs (21), synergizes with interleukin (IL)-1β to enhance cytokine production by GFs (22). In this study, we performed a comprehensive analysis of the interaction between three species of oral bacteria that play distinct roles in periodontitis: the pathobiont *Fusobacterium nucleatum*, the gram-negative pathogen *P. gingivalis*, and the gram-positive pathogen *F. alocis,* and a broad range of inflammatory cytokines in the regulation of COX-2 and PGE2 in GFs. A strong synergy between all tested bacterial species and inflammatory cytokines in COX-2 and PGE2 induction in GFs was noted, with the most prominent synergistic effect observed upon *F. nucleatum* infection. We also show that amplification of PGE2 production by the simultaneous presence of bacteria and cytokines is fibroblast-specific, is mediated by the p38 mitogen-activated protein kinase (MAPK) pathway, and may drive excessive IL-8 production by macrophages, identifying a potential new mechanism responsible for the stromal-immune cross-talk in periodontitis.

## RESULTS

### Oral bacteria and inflammatory cytokines synergistically induce COX-2 expression and PGE2 production in GFs

**Fig 1:**
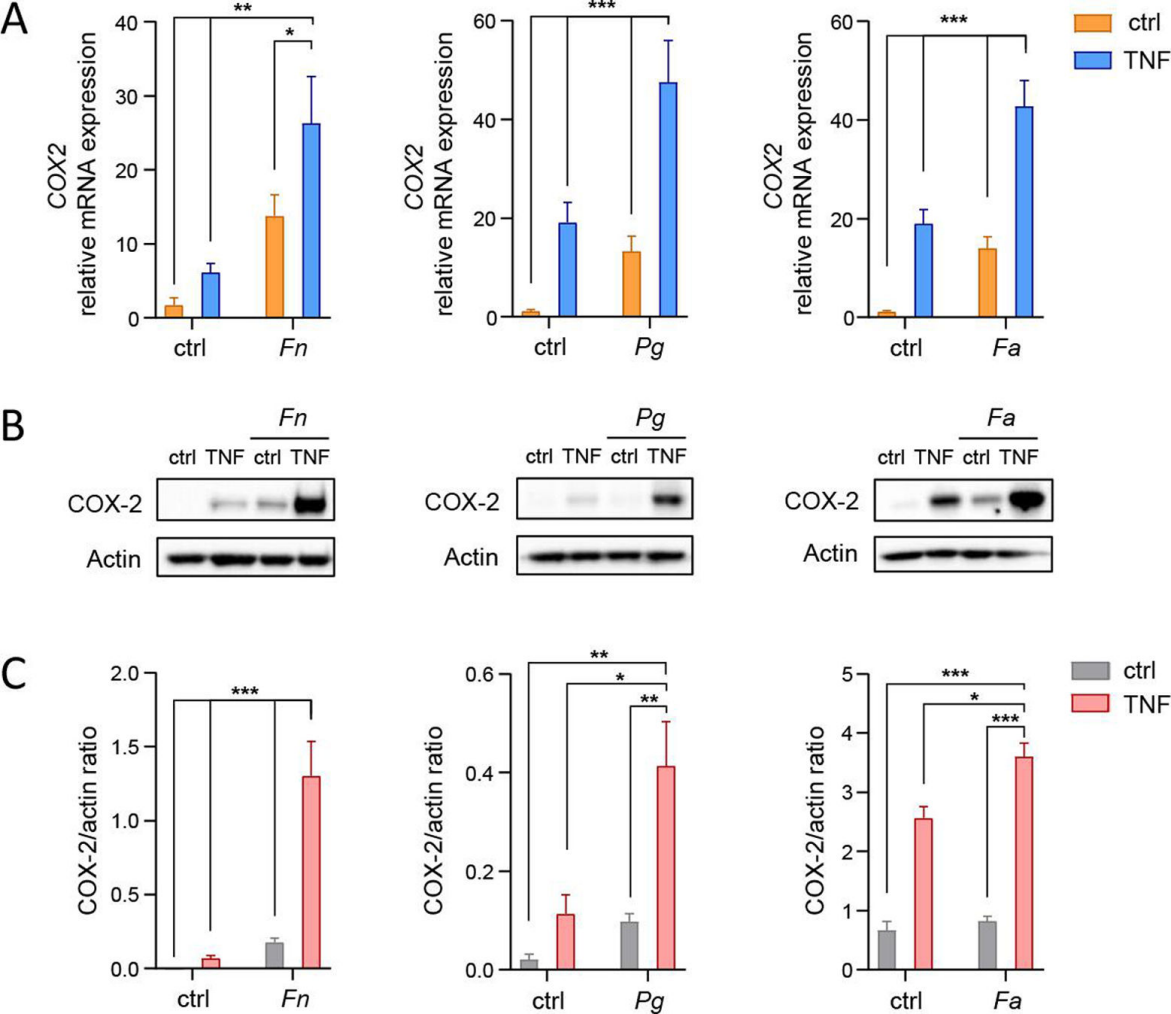
COX-2 expression is synergistically induced by oral bacteria and TNF in GFs. (A) qPCR analysis of *COX-2* expression in GFs infected with *F. nucleatum* (*Fn*) (*n* = 4), *P. gingivalis* (*Pg*) (*n* = 9), or *F. alocis* (*Fa*) (*n* = 8) in the absence (ctrl) or presence of 10 ng/mL TNF for 4 h. (B-C) Western blot analysis of COX-2 levels in GFs treated as in (A) for 24 h. Actin was used as a loading control. (B) Representative blots and (C) results of densitometry analysis (*n* = 3) are shown. Data are presented as mean + SEM. *P < 0.05; **P < 0.01; ***P < 0.001; Two-way ANOVA followed by Tukey multiple comparison test.

To identify potential interactions between oral bacteria and inflammatory cytokines in the regulation of the COX-2-PGE2 axis, we infected GFs with two well-characterized oral pathogens, *P. gingivalis* (ATCC 33277) and *F. alocis* (ATCC 35896), or the pathobiont *F. nucleatum* (ATCC 10953) in the absence or presence of TNF—the cytokine detected in large quantities in the gingival tissue of periodontitis patients (23). We found that each of the tested bacteria amplified TNF-induced COX-2 mRNA expression (Fig. 1A). The interaction between bacteria and TNF translated into even more pronounced differences in COX-2 protein levels: GF stimulation with TNF in the presence of *F. nucleatum*, *P. gingivalis*, and, to a lesser extent, *F. alocis* caused a synergistic induction of COX-2 (Fig. 1B and C; Fig. S1A). Of note, the observed amplification of COX-2 induction was not dependent on the timing of infection and stimulation: a similar degree of COX-2 upregulation was observed in GFs infected with *P. gingivalis* for 2 h prior to washing and subsequent stimulation with TNF and in cells that were subjected to simultaneous infection and stimulation (Fig. S1B). This observation indicates that exposure to an oral pathogen is sufficient to prime GFs for amplified COX-2 induction upon the next inflammatory challenge.

**Fig 2:**
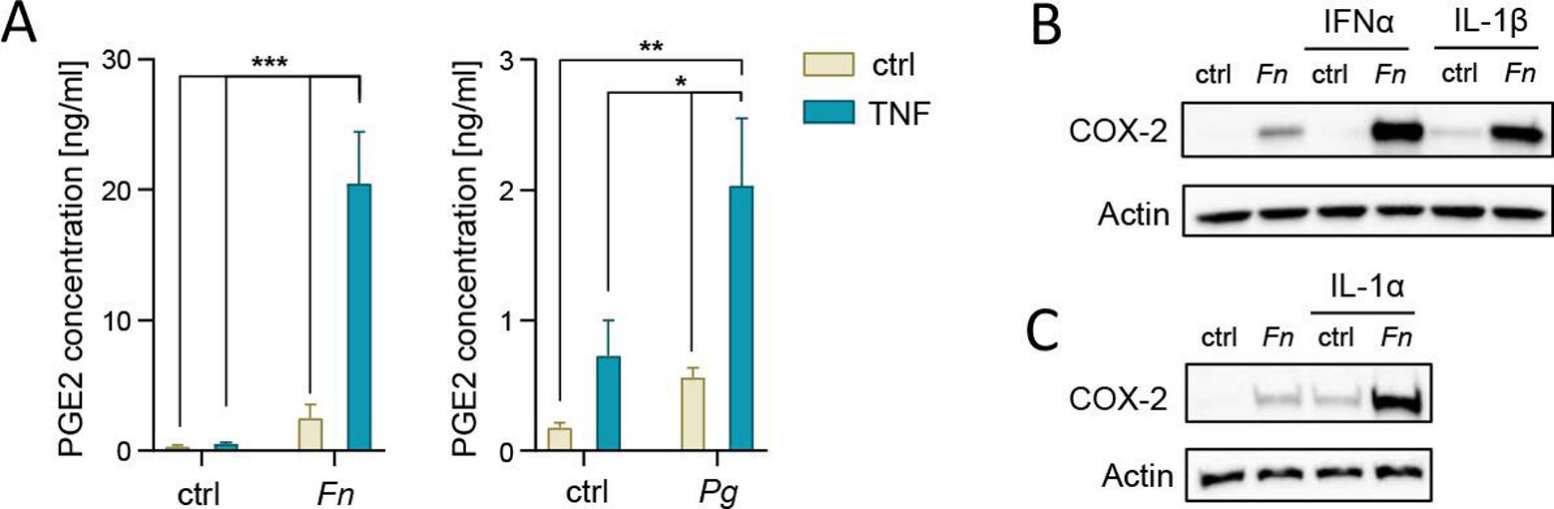
PGE2 production and COX-2 expression are synergistically induced by oral bacteria and inflammatory cytokines in GFs. (A) PGE2 production by GFs infected with *F. nucleatum* (*Fn*) (*n* = 5) or *P. gingivalis* (*Pg*) (*n* = 7) with or without TNF (10 ng/mL) for 24 h. Data are shown as mean + SEM. *P < 0.05; **P < 0.01; ***P < 0.001; two-way ANOVA followed by Tukey multiple comparison test. (B and C) Western blot analysis of COX-2 levels in GFs infected with *F. nucleatum* in the absence (ctrl) or presence of IFNα (100 U/mL), IL-1β (10 pg/mL), or IL-1α (10 pg/mL) for 24 h. Actin was used as a loading control, and results representative of two independent experiments are shown.

Consistent with changes in COX-2 protein levels, we observed synergistic increases in the production of PGE2 in GFs that were simultaneously stimulated with TNF and infected with *F. nucleatum* or *P. gingivalis*, with the former exerting a more prominent effect (Fig. 2A). Because of that, in the majority of subsequent experiments, *F. nucleatum* was used as a model to study the scope, underlying mechanisms, and functional consequences of the synergy between oral bacteria and TNF in the regulation of the COX-2-PGE2 axis.

To verify whether the observed synergistic activation of the COX-2-PGE2 axis is specific for TNF, we analyzed the effect of several cytokines that are involved in periodontitis pathogenesis, including IL-1β, IL-1α, and interferon-α (IFNα) (23). All tested cytokines amplified COX-2 protein accumulation in GFs that were simultaneously infected with *F. nucleatum* (Fig. 2B and C), suggesting that this mode of COX-2 regulation is universal across a broad spectrum of inflammatory mediators and pathogens that GFs interact with in the inflamed gingival tissue during periodontitis.

### Synergistic induction of COX-2 by oral bacteria and inflammatory cytokines is specific for fibroblasts and is dependent on TLR2 activation

**Fig 3:**
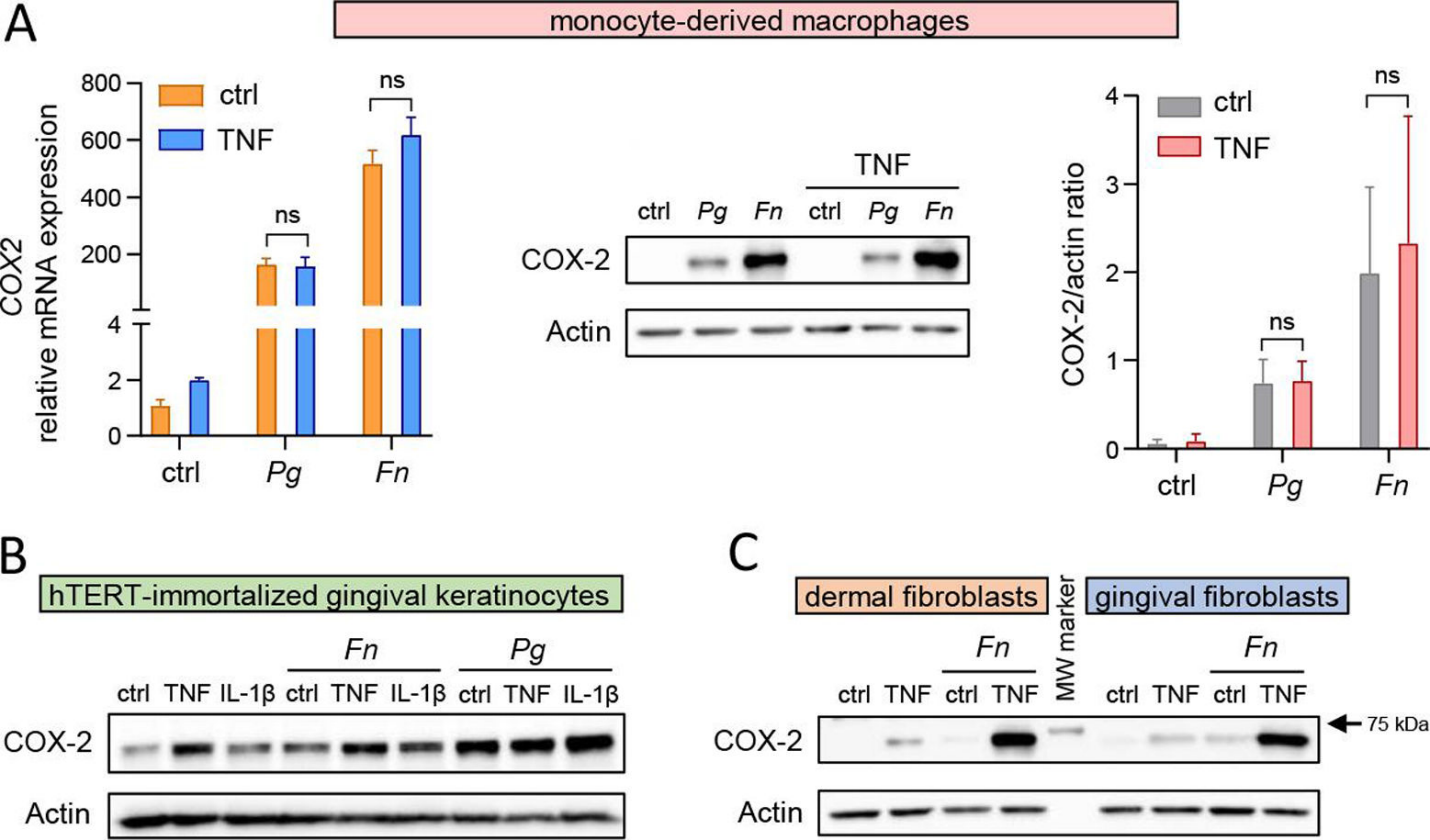
Synergistic induction of COX-2 by oral bacteria and cytokines is specific for fibroblast populations. (A) qPCR analysis of COX-2 expression in MDMs infected with *P. gingivalis* (*Pg*) or *F. nucleatum* (*Fn*) without (ctrl) or with TNF (10 ng/mL) for 4 h (left panel). Western blot analysis of COX-2 in MDMs (*n* = 3) treated as in (A) for 24 h (middle and right panel). Actin was used as a loading control. Representative blots (middle panel) and results of densitometry analysis (right panel) are shown. Data are presented as mean + SEM. ns: not significant; two-way ANOVA followed by Tukey multiple comparison test. (B) Western blot analysis of COX-2 in TIGKs infected with *F. nucleatum* or *P. gingivalis* in the absence (ctrl) or presence of TNF (10 ng/mL) or IL-1β (100 pg/mL) for 24 h. (C) Western blot analysis of COX-2 in DFs and GFs infected with *F. nucleatum* in the absence (ctrl) or presence of TNF (10 ng/mL) for 24 h. (B and C) Actin was used as a loading control, and results representative of two independent experiments are shown.

Next, we tested whether the observed synergistic activation of the COX-2-PGE2 axis by oral pathogens and TNF is unique for GFs or if it represents a general mechanism that is operational in multiple immune and non-immune cell types that are present in the gingival tissue. First, we analyzed COX-2 regulation in macrophages, which are the main immune cell type producing PGE2 in the gingival tissue, using monocyte-derived macrophages (MDMs) as a model. TNF stimulation caused only a minor increase in *COX2* mRNA expression in MDMs and had no significant effect on *COX2* transcript levels induced by *P. gingivalis* and *F. nucleatum* (Fig. 3A; left panel). Consistently, MDM stimulation with TNF did not cause a detectable induction of COX-2 and had no effect on *P. gingivalis*- and *F. nucleatum*-induced COX-2 protein levels (Fig. 3A; middle and right panel). Next, we infected the gingival epithelial cell line TIGK (telomerase- immortalized gingival keratinocytes) with oral bacteria in the presence of inflammatory cytokines, and, similar to MDMs, did not observe any synergy between them in COX-2 induction. Although TNF and *P. gingivalis* alone markedly upregulated COX-2 in TIGKs, the combination of both factors did not result in any further protein accumulation (Fig. 3B). Finally, we compared COX-2 regulation in GFs and dermal fibroblasts (DFs) derived from healthy skin specimens and observed that infection of each cell type with *F. nucleatum* in the presence of TNF results in a nearly identical synergistic induction of COX-2 (Fig. 3C). Although DF exposure to an oral pathobiont does not represent a physiologically relevant model, this experiment demonstrates that synergistic induction of COX-2 by bacteria and TNF is not specific for GFs but rather represents a general feature of fibroblasts regardless of their origin. By contrast, a similar mode of COX-2 regulation is not present in macrophages or epithelial cells.

**Fig 4:**
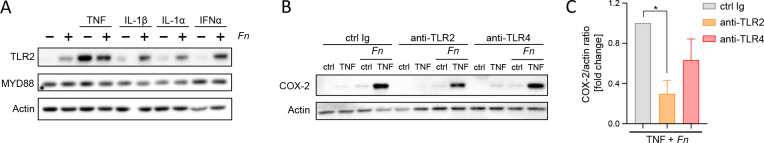
Synergistic induction of COX-2 by *F. nucleatum* and TNF requires TLR2. (A) Western blot analysis of TLR2 and MYD88 in GFs infected with *F. nucleatum* (*Fn*) in the absence or presence of TNF (10 ng/mL), IL-1β (10 pg/mL), IL-1α (10 pg/mL), or IFNα (100 U/mL) for 24 h. (B and C) Western blot analysis of COX-2 in GFs incubated with control Ig, anti-TLR2, or anti-TLR4 antibodies for 30 min prior to infection with *F. nucleatum* with or without TNF (10 ng/mL) stimulation for 24 h. Actin was used as a loading control. (B) Representative blots and (C) results of densitometry analysis (*n* = 3) are shown. Data are presented as mean + SEM. *P < 0.05; one-way ANOVA followed by Bonferroni multiple comparison test.

To gain more insight into the contribution of specific receptors and other molecules involved in pathogen recognition to the synergistic induction of COX-2 by cytokines and bacteria in GFs, we analyzed the mRNA expression profiles of *TLR2*, *TLR4*, *NOD2*, *MYD88*, *CGAS*, and *STING1* in GFs, TIGKs, and MDMs stimulated with TNF, IL-1β, IL-1α, or IFNα. While this screening did not reveal any common regulatory patterns induced in GFs by all the analyzed cytokines, it demonstrated that toll-like receptor-2 (TLR2) has very low basal expression compared to MDMs or TIGKs, but is strongly upregulated by TNF (Fig. S2). Consistently, protein levels of TLR2, but not MYD88, underwent dynamic regulation in GFs that were stimulated with cytokines both in the absence or presence of *F. nucleatum* infection (Fig. 4A). By contrast, TLR2 protein expression was only moderately affected by cytokines and *F. nucleatum* in TIGKs and MDMs (Fig. S3). Based on these observations and given the well-established role of TLR2 in the activation of GFs by oral pathogens (20, 21, 24), we analyzed the involvement of TLR2 and, as a reference, TLR4 in the synergistic upregulation of COX-2. GF pretreatment with a TLR2 neutralizing antibody significantly suppressed COX-2 protein induction in cells simultaneously stimulated with TNF and infected with *F. nucleatum*, whereas application of a TLR4 blocking antibody had a modest effect on COX-2 expression which did not reach statistical significance (Fig. 4B and C).

### p38 MAPK activation drives synergistic induction of the COX-2-PGE2 axis by oral bacteria and inflammatory cytokines

**Fig 5:**
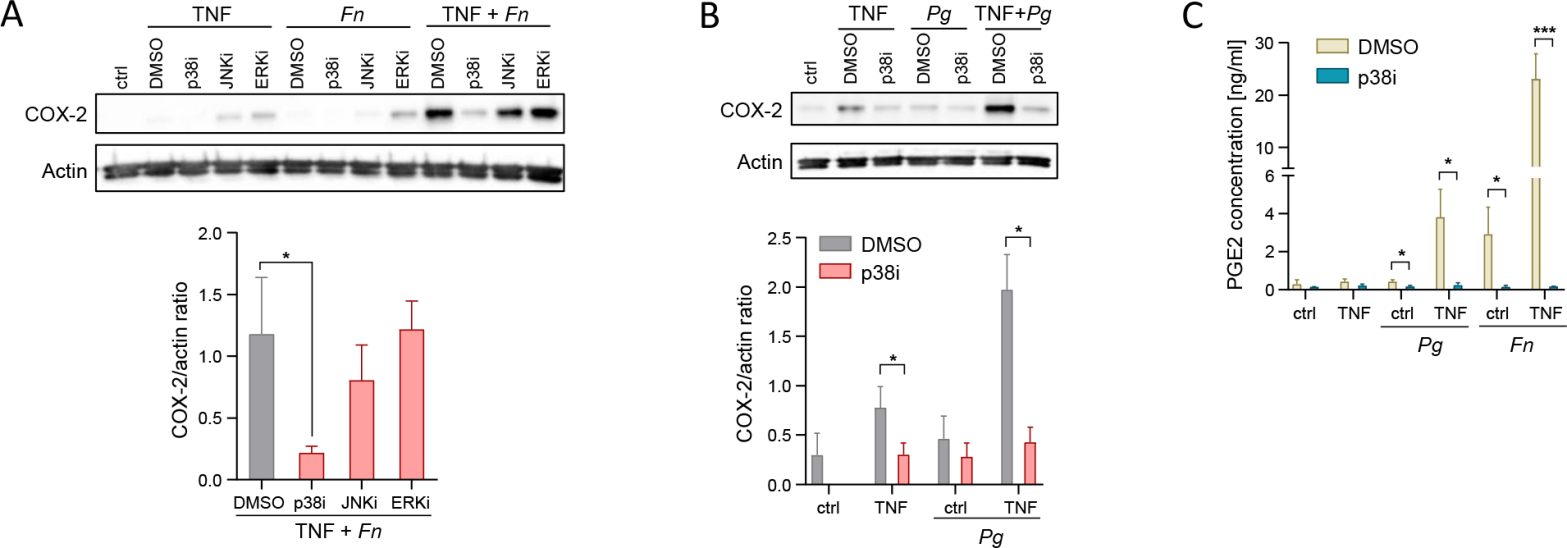
p38 MAPK activation drives synergistic induction of COX-2 and PGE2 by oral bacteria and TNF. (A) Western blot analysis of COX-2 in GFs pretreated with DMSO or MAPK inhibitors (p38i, JNKi, and ERKi) prior to infection with *F. nucleatum* (*Fn*) in the absence (ctrl) or presence of 10 ng/mL TNF for 24 h. Representative blots (upper panel) and results of densitometric analysis for selected conditions (*n* = 3, bottom panel) are shown. *P < 0.05; ratio paired t test. (B) Western blot analysis of COX-2 in GFs pretreated with DMSO or p38 inhibitor (p38i) prior to infection with *P. gingivalis* (*Pg*) without (ctrl) or with stimulation with 10 ng/mL TNF for 24 h. Representative blots (upper panel) and results of densitometric analysis (*n* = 3, bottom panel) are shown. *P < 0.05; One-way ANOVA followed by Bonferroni multiple comparison test. (C) PGE2 production by GFs (*n* = 5) pretreated with DMSO or p38i prior to infection with *P. gingivalis* or *F. nucleatum* in the absence (ctrl) or presence of 10 ng/mL TNF for 24 h. Data are presented as mean + SEM. *P < 0.05; **P < 0.01; ratio paired t test.

To identify the molecular mechanisms underlying synergistic COX-2 induction by oral bacteria and TNF, we analyzed the involvement of MAPK pathway components, many of which are known as important regulators of the COX-2-PGE2 axis. We used a panel of small molecule inhibitors of p38, c-Jun N-terminal kinase (JNK), and extracellular signal-regulated kinase (ERK) and confirmed their selectivity in TNF-stimulated GFs by analyzing phosphorylation of MAPK-activated protein kinase 2 (MAPKAPK2, downstream of p38), c-Jun (downstream of JNK), and ERK (Fig. S4). While the tested MAPK inhibitors had variable effects on COX-2 protein levels induced by *F. nucleatum* or TNF alone, only inhibition of p38 significantly suppressed the synergistic induction of COX-2 in GFs subjected to simultaneous stimulation and infection (Fig. 5A). Similarly, p38 inhibition prevented COX-2 upregulation by TNF in the presence of *P. gingivalis* infection (Fig. 5B). In line with the effects on COX-2 protein induction, the p38 inhibitor almost completely blocked the production of PGE2 by GFs stimulated with TNF during infection with *P. gingivalis* or *F. nucleatum* (Fig. 5C).

### PGE2 secreted by GFs exposed to oral bacteria and cytokines promotes *IL-8* expression in macrophages

**Fig 6:**
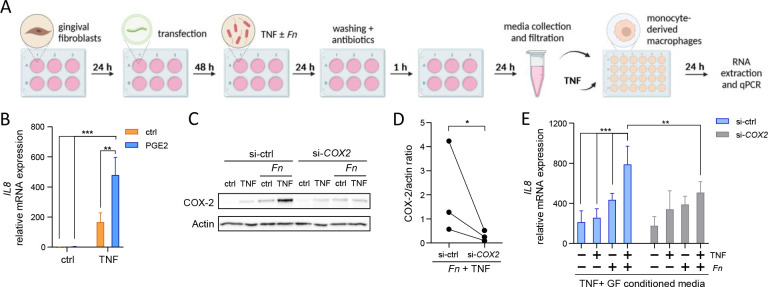
GF-derived PGE2 promotes *IL8* expression in MDMs. (A) Schematic representation of the experimental setup. Created in BioRender. Grabiec, A. (2025) https://BioRender.com/k40a236. (B) qPCR analysis of *IL8* expression in MDMs stimulated with PGE2 in the absence (ctrl) or presence of 10 ng/mL TNF for 24 h. **P < 0.01; ***P < 0.001; Two-way ANOVA followed by Tukey multiple comparison test. (C and D) Western blot analysis of COX-2 in GFs transfected with control non-targeting siRNA (si-ctrl) or COX-2-targeting siRNA (si-COX-2) before infection with *F. nucleatum* (*Fn*) without (ctrl) or with 10 ng/mL TNF for 24 h. Representative blots (upper panel) and results of densitometric analysis for selected conditions (*n* = 3, bottom panel) are shown. *P < 0.05; ratio paired t test. (E) qPCR analysis of *IL8* expression in MDMs after 24 h stimulation with TNF (50 ng/mL) in the presence of conditioned media from GFs (*n* = 4) that were treated as in (C). Data are presented as mean + SEM. *P < 0.05; ***P < 0.001; comparisons between MDMs subjected to GF conditioned media from different transfection conditions were performed using ratio paired t test, whereas comparisons between MDM responses to GF conditioned media from different infection/stimulation conditions were performed using one-way ANOVA followed by Bonferroni multiple comparison test.

In periodontitis-affected gingival tissue, the GF cross-talk with immune cells, in particular monocytes and macrophages, modulates multiple aspects of the immune response against oral pathogens (25, 26). Therefore, to verify the functional consequences of synergistic induction of PGE2 by oral bacteria and TNF in GFs, we established an experimental system where conditioned media from GFs subjected to infection with *F. nucleatum* in the presence or absence of TNF were used as a stimulus for MDMs (Fig. 6A). First, we confirmed that exogenous PGE2 significantly amplifies *IL8* mRNA induction by TNF in MDMs (Fig. 6B), consistent with previous observations in PBMCs (27). To test whether the potential regulation of *IL8* in MDMs by GF-conditioned media is PGE2-dependent, we silenced *COX2* in GFs using siRNA prior to infection and stimulation. COX-2 knockdown efficiency was confirmed by western blot (Fig. 6C and D). When MDMs were stimulated with TNF in the presence of conditioned media from GFs treated with control siRNA, we observed condition-dependent induction of *IL8*: conditioned media from cells infected with *F. nucleatum* in the presence of TNF induced significantly higher expression of *IL8* compared to media from GFs infected with bacteria or stimulated with TNF alone (Fig. 6E). This effect was significantly diminished when MDMs were treated with conditioned media from GFs that lacked *COX2* expression (Fig. 6E), confirming that the observed interplay between GFs and macrophages is partly PGE2-dependent. Collectively, these results identify a potential new mechanism of the immune-stromal cross-talk in periodontitis. This cross-talk is mediated by the synergistic induction of PGE2 in GFs by oral bacteria and the inflammatory tissue environment, leading to the amplification of *IL-8* expression in macrophages.

## DISCUSSION

In recent years, great progress has been made in fibroblast biology, mainly driven by the development of single-cell transcriptomics. The unique roles of specific fibroblast populations in health and disease have been discovered, including the detailed characterization of the contributions of fibroblasts to organ-specific immune responses (28) as well as the identification of specific subsets of synovial and skin fibroblasts involved in rheumatoid arthritis and atopic dermatitis pathogenesis, respectively (29, 30). Not surprisingly, a similar heterogeneity of fibroblast populations was recently demonstrated in the human oral barrier tissues (31). Strikingly, the comparison of the healthy and periodontitis-affected oral mucosa at the single-cell level revealed the central role of GFs in neutrophil recruitment to the inflamed gingival tissue (3). Here, we identify a new mechanism responsible for the cross-talk between GFs and neutrophils. This mechanism involves the synergistic induction of the COX-2-PGE2 axis by oral bacteria and inflammatory cytokines, which could enhance the production of the neutrophil chemokine IL-8 by macrophages.

Transcription of the *PTGS2* gene is dynamically regulated by a plethora of host-derived and external factors that activate distinct receptors and intracellular signaling pathways, the interaction of which may result in robust changes in COX-2 expression. In peripheral blood macrophages, COX-2 was synergistically upregulated by CD40 ligation in the presence of monocyte chemoattractant protein-1, leading to PGE2-dependent enhancement of vascular endothelial growth factor production (32). In lung fibroblasts and vascular smooth muscle cells, IL-1β synergized with angiotensin II in COX-2 induction (33, 34). Similarly, simultaneous stimulation with TNF and IL-1β caused a synergistic induction of COX-2 and PGE2 in GFs (35). Interestingly, exogenous PGE2 enhanced TNF-induced COX-2 and mPGES-1 expression in GFs, indicating that PGE2 production is regulated by a positive feedback loop which may also involve autocrine responses to PGE2 secreted by activated GFs (16). A limited number of reports also indicate that COX-2 may be synergistically induced by bacterial virulence factors and host-derived mediators. Simultaneous stimulation of human intestinal myofibroblasts with LPS from *Escherichia coli* and IL-17 enhanced COX-2 expression and PGE2 release, which was associated with synergistic amplification of NF-κB DNA-binding activity (36). In dental pulp fibroblasts, COX-2 was synergistically upregulated by TLR2 ligation and histamine (37). However, to the best of our knowledge, our study provides the first evidence for synergistic interaction between live bacteria and inflammatory cytokines in the induction of the COX-2-PGE2 axis that is specific for fibroblast populations. Our data also indicate that among the receptors involved in the recognition of oral pathogens by GFs, TLR2 engagement is essential for the synergy between *F. nucleatum* and TNF in COX-2 induction. This is consistent with previous studies demonstrating the key role of TLR2 in host cell activation by *F. nucleatum* (38). Although we did not assess the contribution of TLRs to the synergy between TNF and *P. gingivalis* or *F. alocis*, it is noteworthy that both pathogens are also known to activate TLR2 (18, 21).

Despite the prominent role of PGE2 in periodontitis pathogenesis, direct targeting of COX-2 does not appear to be a promising therapeutic strategy. Nonsteroidal anti-inflammatory drugs (NSAIDs) targeting either both COX isoforms or selective COX-2 inhibitors have been tested in several clinical trials as an adjunct to conventional non-surgical periodontal therapy. They uniformly caused significant, albeit small, improvement in clinical parameters of disease activity and reduction in alveolar bone loss (39, 40). However, the long-term use of these compounds is associated with a high rate of side effects, including serious cardiovascular adverse events, precluding their broad application as anti-inflammatory adjunctive therapy in periodontitis (41). Instead, excessive PGE2 production by GFs can be modulated by targeting upstream regulators of COX-2 expression, and our data indicate a central role for p38 MAPK in the stimulation of the COX-2-PGE2 axis. This observation is in line with other studies demonstrating that p38 activation is required for synergistic COX-2 induction in dental pulp fibroblasts (37) and lung fibroblasts (34), though other pathways are also involved in the complex regulation of COX-2 expression (35, 42). Among MAPKs, p38 phosphorylation levels correlated with clinical parameters of periodontal disease progression, and oral administration of a selective p38α inhibitor protected against alveolar bone loss in LPS-induced periodontitis in rats (43, 44). The therapeutic effect of p38 inhibition was associated with diminished inflammatory cytokine levels and reduced numbers of osteoclasts in the gingival tissue, though the regulation of COX-2 or PGE2 was not assessed in this model (44). It should, however, be noted that the therapeutic potential of p38 inhibitors was thoroughly evaluated in clinical trials in many inflammatory diseases, in particular rheumatoid arthritis, showing limited efficacy and unacceptable safety profiles (45). Despite the failure of direct p38 targeting in the clinic, it is likely that targeting other components of this pathway, such as the upstream MAPK kinases (MKK3 and MKK6) or the downstream p38 target MAPKAPK2 could result in a more favorable clinical outcome (46). It remains to be tested whether inhibition of other components of this pathway mimics the suppressive effect of p38 inhibition on the synergistic induction of PGE2 by bacteria and cytokines in GFs.

The ability of PGE2 to enhance IL-8 production is well documented. Stimulation with PGE2 upregulated IL-8 in pulmonary microvascular endothelial cells (47), airway and colonic epithelial cells (48, 49), as well as lung fibroblasts (50), suggesting that excessive PGE2 production may contribute to neutrophilic inflammation in many organs. This effect is not restricted to non-immune cells since PGE2 also enhanced TNF-mediated *IL8* induction in monocytic cell lines and peripheral blood mononuclear cells (PBMCs) (27). PGE2-induced enhancement of *IL8* expression was predominantly driven by the engagement of the EP4 receptor, involving both transcriptional and posttranscriptional regulatory mechanisms (47, 48, 51). Our results extend those observations, demonstrating that GF-derived PGE2 amplifies TNF-induced *IL8* expression in differentiated MDMs, providing a functional link between stromal cell activation and modulation of macrophage functions which is PGE2-dependent and may lead to enhanced neutrophil recruitment.

Apart from its potential effect on neutrophil recruitment, synergistic induction of PGE2 by oral pathogens and cytokines may contribute to periodontitis pathogenesis by other mechanisms. Numerous reports have suggested that PGE2 promotes alveolar bone resorption through the regulation of receptor activator of nuclear factor-κ B ligand (RANKL) and osteoprotegerin (OPG) ratio that would favor osteoclast formation (52, 53). Notably, an agonist of the PGE2 receptor EP4 was sufficient to induce bone resorption in mouse calvaria cultures (54), whereas the NSAID indomethacin suppressed lipoteichoic acid-induced bone resorption in *ex vivo* cultures of mouse alveolar bone (55). In animal models of LPS-induced bone resorption, administration of indomethacin or an EP4 receptor antagonist as well as deletion of the *mPges1* gene suppressed osteoclastogenesis and/or protected against alveolar bone loss (14, 56). However, it is important to note that a vast majority of the reports demonstrating the role of PGE2 in osteoclastogenesis were based on animal models or culture systems using animal cells. Although clinical studies of NSAIDs in periodontitis patients indicate that COX-2 inhibition may have protective effects on bone damage (40), experimental evidence directly showing the functional role of the COX-2-PGE2 axis in bone resorption in humans is lacking. The accumulation of PGE2 in the inflamed gingival tissue could also contribute to the immunopathology of periodontitis through its immunomodulatory roles. PGE2 has been shown to reduce the abilities of macrophages and neutrophils to kill bacteria mainly through inhibition of phagocytosis and suppression of reactive oxygen species generation (57). For example, PGE2 suppressed the phagocytosis of *Klebsiella pneumoniae* and *E. coli* by alveolar macrophages and decreased uptake and killing of *Listeria monocytogenes* by neutrophils (58, 59). In oral epithelial cells, activation of the COX-2-PGE2 axis by *Staphylococcus aureus* promoted the growth and adherence of the pathogen (60). The influence of PGE2 on adhesion, phagocytosis, and elimination of oral pathogens by immune and stromal cells of the periodontium was not formally tested and should be evaluated in future studies.

While the detailed characterization of the biological consequences of synergistic PGE2 induction by oral pathogens and inflammatory cytokines requires further functional studies, our data and the available literature suggest that enhancement of PGE2 levels in gingival tissue could represent another mechanism by which oral pathogens, such as *P. gingivalis*, manipulate the immune response. The suppressive effect of PGE2 on bacterial phagocytosis by host cells could prevent the elimination of pathogens by the immune system while promoting osteoclastogenesis and maintaining the influx of activated immune cells that drive inflammatory tissue breakdown, in particular neutrophils, providing nutrients for inflammophilic bacteria. The results presented here not only identify a new potentially pathogenic role of GFs and highlight their important role as “non-classical” components of the immune system of the oral mucosa but also suggest that targeting the pathways that are essential for synergistic COX-2 and PGE2 induction in GFs could be clinically beneficial as an adjunctive anti-inflammatory host modulation therapeutic option in the treatment of periodontitis.

## MATERIALS AND METHODS

### Subjects, cell isolation, and culture

Gingival tissue specimens were obtained from healthy individuals undergoing the surgical phase of orthodontic treatment at the Chair of Oral Surgery, Faculty of Medicine, Jagiellonian University Medical College, Kraków, Poland. Skin samples were collected from marginal noncancerous skin tissue from patients undergoing surgery for suspected or confirmed cancer at the 2nd Department of General Surgery, Jagiellonian University Medical College, Kraków, Poland. Primary GFs were isolated and cultured as previously described (61), and an identical protocol was used for DF isolation and culture. TIGKs (RRID:CVCL_M095) were kindly provided by Prof. Richard J. Lamont (University of Louisville School of Dentistry) and were cultured in EpiGRO Human Epidermal Keratinocyte Complete Culture Medium (Merck-Millipore). One day prior to and during experiments, GFs and DFs were cultured in antibiotic-free DMEM (VWR 392-0415) containing 2% FBS (Sigma-Aldrich), whereas TIKGs were cultured in the antibiotic-free EpiGRO medium.

Peripheral blood from de-identified healthy individuals was purchased from the Regional Blood Donation and Transfusion Center (Kraków, Poland). Monocytes were isolated from peripheral blood and differentiated into MDMs by 7-day culture in the presence of 50 ng/mL macrophage colony-stimulating factor (M-CSF) (BioLegend) as described previously (21, 62). During experiments, MDMs were cultured in antibiotic-free RPMI (VWR 392-0427) containing 2% FBS.

### Bacterial culture

*P. gingivalis* ATCC 33277, *F. nucleatum* ATCC 10953 or ATCC 25586, and *Filifactor alocis* ATCC 35896 were grown anaerobically on blood agar plates (brain–heart infusion [Becton–Dickinson] with yeast extract containing 0.5 mg/mL L-cysteine, 10 µg/mL hemin and 0.5 µg/mL vitamin K; agar used for *F. alocis* culture was additionally supplemented with 17.42 mg/mL arginine) for 5–7 days at 37°C. *P. gingivalis* and *F. nucleatum* suspensions at optical density (OD)_600_= 1 were prepared as described before (63), whereas *F. alocis* were inoculated into a medium prepared based on Cho et al. with modifications (64) (Table S1) and cultured for 5 days prior to preparation of bacterial suspension at OD_600_ = 1.

### RNA isolation and quantitative (q)PCR

GFs, TIGKs, and MDMs were infected with *P. gingivalis* (MOI 20), *F. nucleatum* (MOI 20), or *F. alocis* (MOI 100) in the presence or absence of 10 ng/mL TNF, 10 pg/mL IL-1β, 10 pg/mL IL-1α, or 100 U/mL IFNα (all from BioLegend) for 4 h. Alternatively, MDMs were treated with 100 nM PGE2 with or without 50 ng/mL TNF for 24 h or were exposed to conditioned media from GFs (1:1 ratio) in the presence of 50 ng/mL TNF for 24 h. Total RNA was extracted using the ExtractMe Total RNA Isolation Kit (Blirt, Gdansk, Poland) and subsequently quantified with a BioPhotometer D30 (Eppendorf). RNA was converted to cDNA using the High-Capacity cDNA Reverse Transcription Kit (Applied Biosystems). qPCRs were conducted on a CFX96 Touch Real-Time PCR Detection System (Bio-Rad), using the PowerUp SYBR Green PCR mix (Applied Biosystems). Sequences of the primers (purchased from Merck) used in the study are listed in Table S2. Data analysis was performed using the CFX Manager software (Bio-Rad). Relative mRNA expression levels were determined using the ΔΔCt method unless indicated otherwise.

### Western blotting

GFs, TIGKs, and MDMs were infected with *P. gingivalis*, *F. nucleatum*, or *F. alocis* (all at MOI 20) in the presence or absence of cytokines (TNF, IL-1β, IL-1α, or IFNα) for 24 h. In some experiments, GFs were pretreated for 1 h with MAPK inhibitors SP600125 (JNK inhibitor; 10 µM), SB203580 (p38 inhibitor; 10 µM), or U0126 (MEK/ERK inhibitor; 10 µM) (all from Cayman Chemical) prior to stimulation with TNF and/or infection with *F. nucleatum*. To study the role of TLRs, GFs were incubated with 2 µg/mL anti-TLR2, or anti-TLR4 neutralizing antibodies or control Ig (all from Invivogen) for 30 min prior to stimulation and infection. Proteins were then extracted by cell lysis in Laemmli’s buffer, resolved by electrophoresis, transferred to PVDF membranes, washed, and blocked as described before (24). The membranes were then incubated at 4°C o/n with primary antibodies specific for COX-2 (#12282), phospho-ERK (#9101), phospho-MAPKAPK2 (#3007), phospho-cJun (#9261), MYD88 (#4283), β-actin (#4967) α-tubulin (#2144) (all from Cell Signaling Technology), or TLR2 (JM22-41, Invitrogen), followed by washing and incubation with horseradish peroxidase (HRP)-conjugated anti-rabbit Ig secondary antibodies (Dako). Blots were developed using a ClarityWestern ECL Substrate and visualized using a ChemiDocMP Imaging System and the ImageLab software (all from BIO-RAD).

### PGE2 ELISA

GFs cultured in 96-well plates (2.5 × 10^4^ cells per well) were infected with *P. gingivalis* (MOI 20) or *F. nucleatum* (MOI 20) in the presence or absence of TNF (10 ng/mL). In some experiments, GFs were preincubated with 10 µM SB203580 prior to infection and stimulation. After 24 h, cell-free supernatants were collected, and the level of PGE2 was determined using Prostaglandin E2 Express ELISA Kit (Cayman Chemical).

### Transfection with siRNA and preparation of conditioned media

GFs were seeded in 12-well plates (1.25 × 10^5^ cells per well) in antibiotic-free DMEM containing 10% FBS. After 24 h, the medium was replaced with Opti-MEM Reduced Serum Medium (Thermo Scientific). Control non-targeting siRNA or COX-2-targeting siRNA (Dharmacon) (final concentration 20 nM) was mixed with DharmaFECT1 (Dharmacon) and Opti-MEM. After incubation for 20 min at RT, the mixtures were used for transfection. Following 24 h of transfection, the medium was replaced with DMEM with 10% FBS, and transfected cells were cultured for an additional 24 h. Subsequently, the medium was changed to antibiotic-free DMEM with 2% FBS, and the cells were infected with *F. nucleatum* (MOI 20) in the presence or absence of 10 ng/mL TNF for 24 h. Cells were then washed with PBS, treated with antibiotics (2.5 mg/mL gentamicin and 2 mg/mL metronidazole) for 1 h, washed again with PBS, and cultured for 24 h in RPMI with 2% FBS. Supernatants were collected, sterile-filtered (0.22 µm), and used for MDM stimulation.

### Statistical analysis

Data are presented as the mean + SEM. The values of “n” refer to independent experiments performed on GFs, DFs, or MDMs from different donors. Comparisons between groups were performed using two-way ANOVA, one-way ANOVA, or ratio paired t-test, where appropriate. *P* values < 0.05 were considered statistically significant.

## Data Availability

The original contributions presented in the study are included in the article and supplemental material. Raw data are available from the corresponding author upon request.

## SUPPLEMENTAL MATERIAL

Supplemental figures and tables mbio.00046-25-s0001.pdf Tables S1 and S2 and Figures S1 to S4.

ASM does not own the copyrights to Supplemental Material that may be linked to, or accessed through, an article. The authors have granted ASM a non-exclusive, world-wide license to publish the Supplemental Material files. Please contact the corresponding author directly for reuse.
