# Supplementary material for: Synergistic induction of PGE2 by oral pathogens and TNF promotes gingival fibroblast-driven stromal-immune cross-talk in periodontitis
Source: mBio. 2025 Apr 3;16(5):e00046-25. doi: 10.1128/mbio.00046-25 (PMC12077116; doi:10.1128/mbio.00046-25)
Supplement: Supplemental figures and tables — Tables S1 and S2 and Figures S1 to S4. [file mbio.00046-25-s0001.pdf]

Supplementary Material

Supplementary table S1. Composition of *F. alocis* culture medium

Component	Amount per L
Beef extract	5 g
Yeast extract	5 g
Bio-tryptone	15 g
Glucose	2 g
L-cysteine	0.6 g
Arginine	17.42 g
Sodium chloride	5 g
Magnesium sulfate	0.1 g
Potassium phosphate dibasic	6 g
Sodium carbonate	0.6 g
L-cysteine	0.5 g
hemin	10 mg
Vitamin K	0.5 mg

Supplementary table S2. Sequences of primers used for qPCR analyses.

Gene	Forward primer	Reverse primer
COX2	AGCCCTTCCTCCTGTGCCT	AATCAGGAAGCTGCTTTTTACCT
IL8	GCTCTGTGTGAAGGTGCAGT	CCAGACAGAGCTCTCTTCCA
TLR2	CGGAATGTCACAGGACAGCA	TACCACAGGCCATGGAAACG
TLR4	ACCATCATTGGTGTGTCGGTC	AGCCAGCAAGAAGCATCAGG
MYD88	CTAAGAAGGACCAGCAGAG	GAAGCATCAGTAGGCATCA
NOD2	GATTGGCTGCCTTCCTTCTA	GAGCGTCTCTGCTCCATCAT
CGAS	GCCAGTAGTGCTTGGTTTCC	GTTCCCCGAAAGAAGAATCC
STING	TCAAGGATCGGGTTTACAGC	TGGCAAACAAAGTCTGCAAG
RPLP0	GCGTCCTCGTGGAAGTGACATCG	TCAGGGATTGCCACGCAGGG

Supplementary Figures

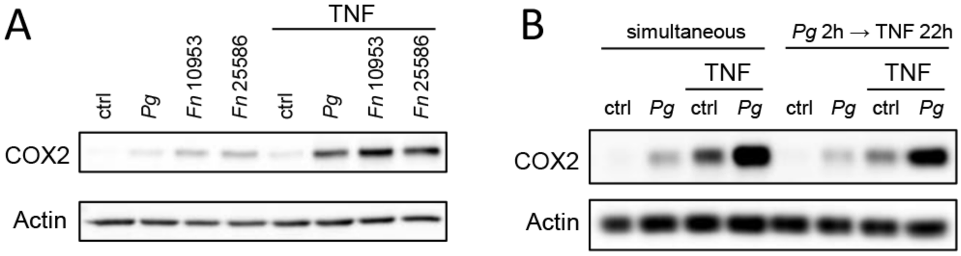

Supplementary figure S1. (A) Western blot analysis of COX-2 in GFs infected with *P. gingivalis* (*Pg*) or two different strains of *F. nucleatum* (*Fn*) (ATCC 10953 or ATCC 25586) in the absence (ctrl) or presence of 10 ng/ml TNF for 24 h. (B) Western blot analysis of GFs that were subjected to two different protocols of *P. gingivalis* (*Pg*) infection and TNF stimulation: cells were either infected and stimulated simultaneously or were infected with *P. gingivalis* for 2 h, followed by washing and stimulation with 10 ng/ml TNF in DMEM containing 2% FBS and antibiotics (2.5 mg/ml gentamicin and 2 mg/ml metronidazole) for 24 h. Actin was used as loading control and results shown are representative of three independent experiments.

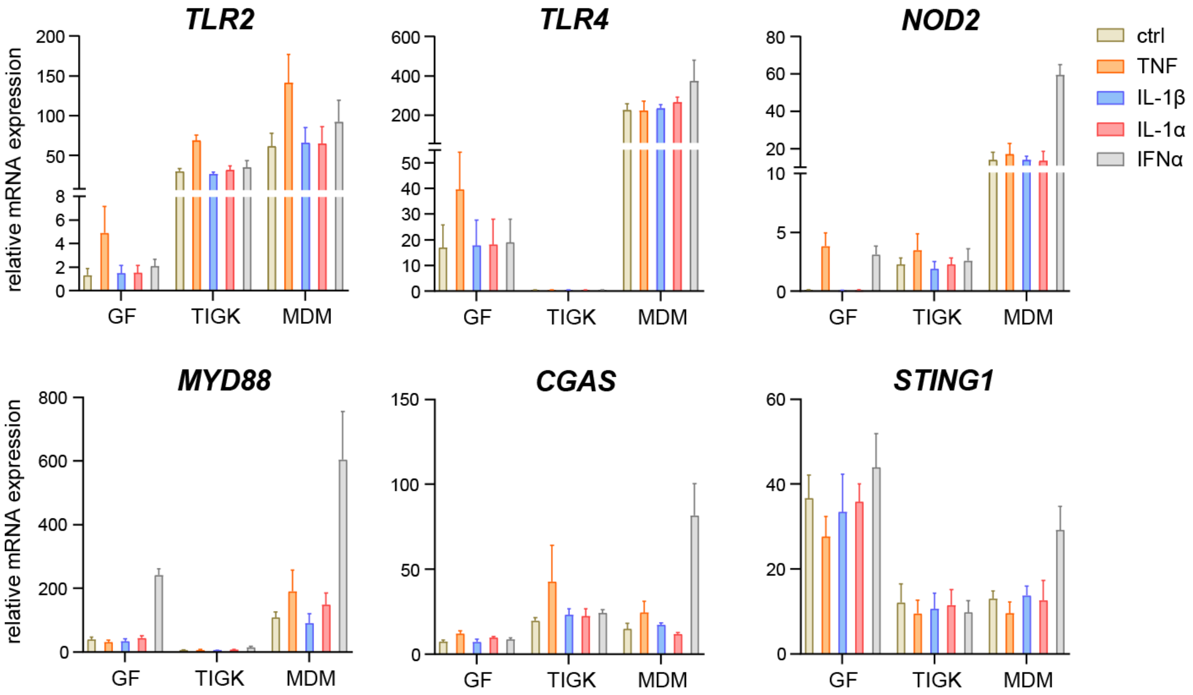

Supplementary figure S2. qPCR analysis of *TLR2*, *TLR4*, *NOD2*, *MYD88*, *CGAS*, and *STING1* expression in GFs, MDMs, and TIGKs (n=3) stimulated with TNF (10 ng/ml), IL-1β (10 pg/ml), IL-1α (10 pg/ml) or IFNα (100 U/ml) for 4 h. Data are shown as mean relative mRNA expression calculated using the ΔCt method to allow for comparison between different cell types.

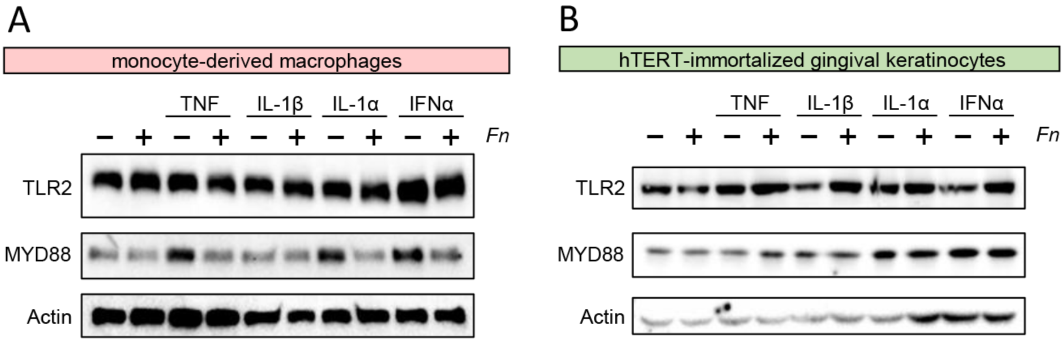

Supplementary figure S3. Western blot analysis of TLR2 and MYD88 in (A) MDMs and (B) TIGKs infected with *F. nucleatum* (*Fn*) in the absence of presence of TNF (10 ng/ml), IL-1β (10 pg/ml), IL-1α (10 pg/ml) or IFNα (100 U/ml) for 24 h. Actin was used as loading control and results representative of two independent experiments are shown.

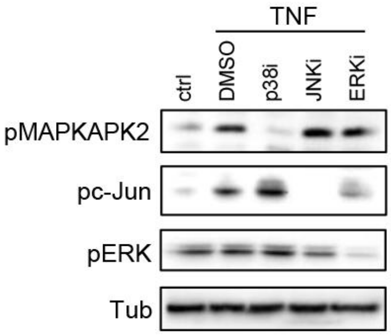

Supplementary figure S4. Western blot analysis of phospho (p)MAPKAPK2, pc-Jun, and pERK in GFs pretreated with MAP kinase inhibitors (p38i, JNKi, ERKi) for 60 min prior to stimulation with 10 ng/ml TNF for 30 min. Tubulin was used as loading and results shown are representative of two independent experiments.
